# The complete mitochondrial genome of *Medicago truncatula*

**DOI:** 10.1080/23802359.2016.1144087

**Published:** 2016-02-10

**Authors:** Changwei Bi, Xuelin Wang, Yiqing Xu, Suyun Wei, Yi Shi, Xiaogang Dai, Tongming Yin, Ning Ye

**Affiliations:** a College of Information Science and Technology, Nanjing Forestry University, Nanjing, Jiangsu, China;; b School of Computer Science and Engineering, Southeast University, Nanjing, Jiangsu, China;; c College of Forest Resources and Environment, Nanjing Forestry University, Nanjing, Jiangsu, China

**Keywords:** Legumes, *Medicago truncatula*, mitochondrial genome

## Abstract

The complete mitochondrial genome of *Medicago truncatula* (*M. truncatula*) was reported in this study. The mitochondrial genome (mitogenome) was assembled to 271 618 nt. The mitogenome contains 31 protein-coding genes, three rRNA genes and 16 tRNAs. The overall base composition of the mitogenome in descending order is A: 27.21%, C: 22.61%, G: 22.78% and T: 27.40%, and the G + C content is 45.39%. Additionally, 30 exons and 17 introns were identified in eight genes and nine tandem repeats were identified with the period size from 10 nt to 33 nt. Phylogenetic analysis shows that the *M. truncatula* genome is evolutionarily closest to that of Lotus japonicas. With the complete mitogenome of *M. truncatula*, it is beneficial to the further research of mitogenome of seed plants, and especially helpful for elucidating vital activities of legumes.

*Medicago truncatula* is originated in the Mediterranean, and it has been widely distributed in other parts of the world with European immigrants. *Medicago truncatula* can be studied as a model organism for legume biology because of its small diploid genome, rapid generation time, prolific seed production and sequenced genome (Young et al. 2011). The complete mitochondrial genome of *M. truncatula* is very important for elucidating vital activities of legumes.

In this study, the complete mitochondrial DNA sequence of *M. truncatula* (accession no. KT971339) was assembled into a circular-mapping genome of lengths 271 618 nt by Newbler3.0 (Iorizzo et al. 2012), which is smaller than some other legumes, such as *Glycine max* (402.5 kb) and *Vigna radiata* (401.2 kb). The sample strain A17 was acquired from the Genetic Resource Centre, SARDI (South Australian Research and Development Institute, Adelaide, South Australia 5001) (Kamphuis et al. 2007), and the tissue was also stored there. We obtained the sequencing data from NCBI (BioSample: SAMN02299339). The G + C content of the mitogenome is 45.39%, which is nearly the same as other legumes (Alverson et al. 2011; Chang et al. 2013). In order to exhibit the mitogenome better, we build a GBrowse for the mitogenome (http://bio.njfu.edu.cn/gb2/gbrowse/Medicago_truncatula_mt/).

Using BLAST and tRNA scan-SE (Alverson et al. 2010), 50 genes (63 333 nt in total length) were identified on the mitogenome of *M. truncatula*, including 31 protein-coding genes, three rRNA genes and 16 tRNA genes. Also, 30 exons and 17 introns were identified in eight genes (nad1, 2, 4, 5, 7, ccmFc, rps3 and rps10). Most of the protein-coding genes have the common start codon: ATG, except *nad4L*, *nad1* and *mttB*, which use ACG and ACT. Four types of stop codons are identified in the protein-coding genes: TAA (14 genes: *rps1*, *rps1*, *nad9*, *rps3*, *rpl16*, *rpl5*, *nad2*, *nad4L*, *atp4*,* nad6*, *rps4*, *atp8*, *nad1* and *atp9*), TGA (10 genes: *ccmB, ccmC, ccmFn, cob, mttB, rps10, cox3, nad4, rps12* and *atp6*), TAG (four genes: *atp1, rps14, matR* and *nad7*) and CGA (ccmFc; C to U RNA editing on the first site). The stop codon, CGA, is also observed in *Vigna radiate* (Alverson et al. 2011).

Comparing the *M. truncatula* mitogenome with itself, we found some repeats in it. Repeats contribute very little to the whole mitogenome (6541 nt, 2.41%), which is similar to *Vigna radiata* (2.7%). Most repeats are less than 100 nt in length, and most of these are ranged from 20 to 60 in length. Additionally, nine tandem repeats are identified by Tandem Repeats Finder with the period size from 10 nt to 33 nt (Benson & Benson 1999). A neighbour-joining analysis was applied to the 30 plant mitogenomes based on amino acid sequences of 21 protein-encoding genes (*atp1*, *atp4*, *atp8*, *atp9*, *ccmB*, *ccmC*, *ccmFc*, *cob*, *cox1*, *cox3*, *matR*, *nad1*, *nad2*, *nad3*, *nad4*, *nad4L*, *nad5*, *nad6*, *nad7atp1*, *atp4*, *atp8*, *atp9*, *ccmB*, *ccmC*, *ccmFc*, *cob*, *cox1*, *cox3*, *matR*, *nad1*, *nad2*, *nad3*, *nad4*, *nad4L*, *nad5*, *nad6*, *nad7* and *nad9*). The phylogenetic tree of these mitogenomes shows that the *M. truncatula* mitogenome is evolutionarily closest to that of *Lotus japonicas* (Figure 1). These results presented here contribute to the further biological study into the mitogenome of seed plants and provide important information on evolutionary of seed plants.

**Figure 1.**
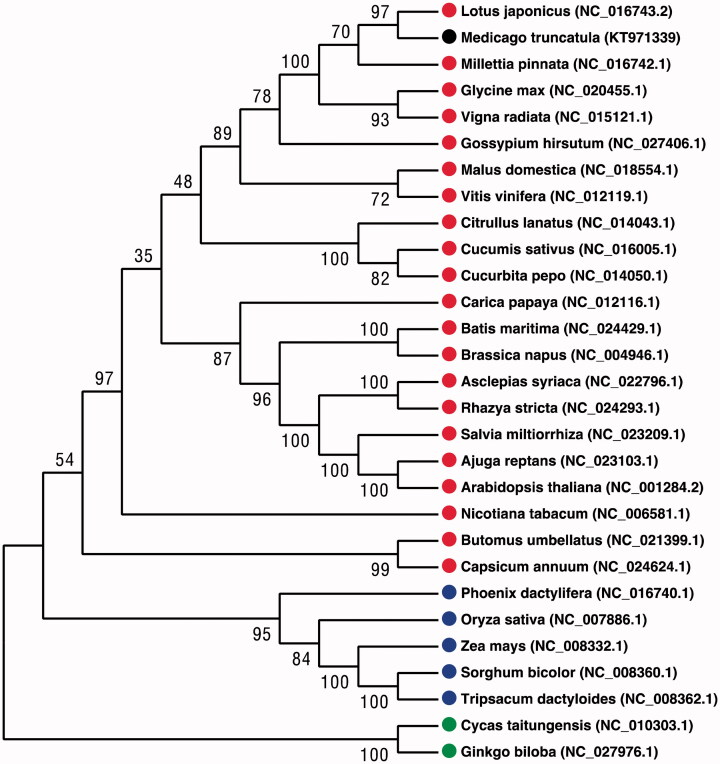
Neighbour-joining method of 29 representative plant mitogenomes using the coding sequences of 21 genes (*atp1*, *atp4*, *atp8*, *atp9*, *ccmB*, *ccmC*, *ccmFc*, *cob*, *cox1*, *cox3*, *matR*, *nad1*, *nad2*, *nad3*, *nad4*, *nad4L*, *nad5*, *nad6*, *nad7* and *nad9*). Numbers above each node represent bootstrap values from 1000 replicates. Black and red circles indicate Magnoliopsida, blue circles indicate Liliopsida and green circles indicate Gymospermae.
